# Supplementary material for: Lymph node volume predicts survival in esophageal squamous cell carcinoma treated with neoadjuvant chemoradiotherapy and surgery
Source: PLoS One. 2024 Mar 28;19(3):e0300173. doi: 10.1371/journal.pone.0300173 (PMC10977715; doi:10.1371/journal.pone.0300173)
Supplement: S1 Fig — (A) The cross-sectional view of primary tumor (red color) and LN (orange color). (B)The coronal view of primary tumor (red color) and LN (orange color). (C)The 3D construction for volume measurement of primary tumor (red color) and LN (orange color). (PDF) [file pone.0300173.s001.pdf]

Supporting information

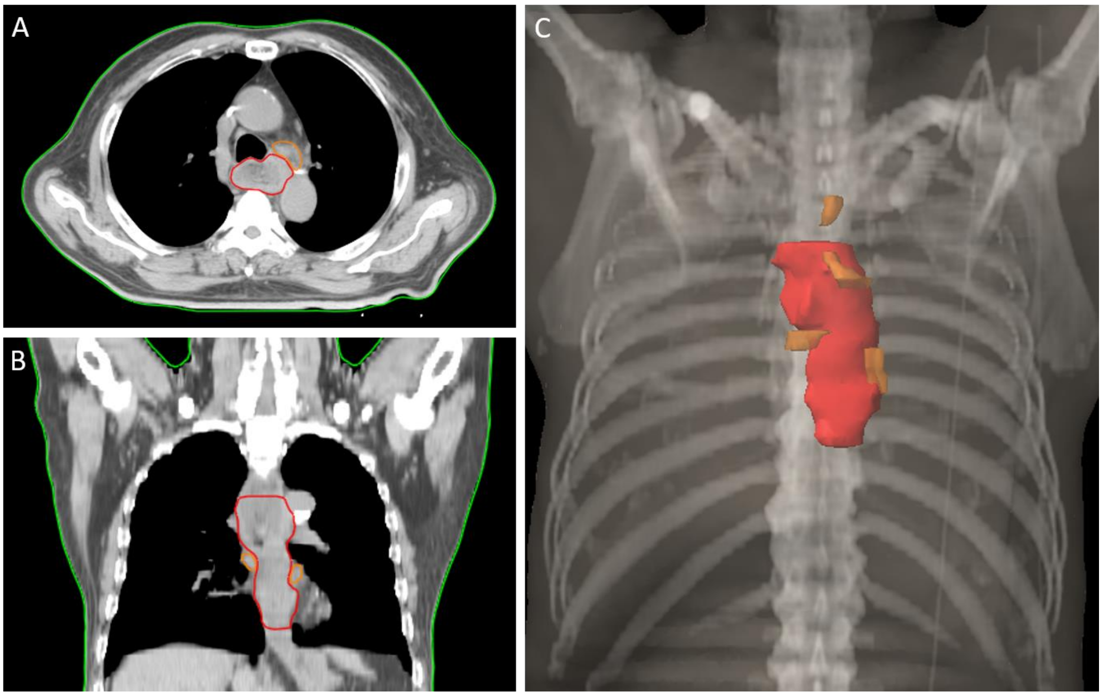

S1 Fig. The representative images of primary tumor and LN delineation and volume measurement. (A) The cross-sectional view of primary tumor (red color) and LN (orange color). (B) The coronal view of primary tumor (red color) and LN (orange color). (C) The 3D construction for volume measurement of primary tumor (red color) and LN (orange color).
