# Supplementary material for: Lymph node volume predicts survival in esophageal squamous cell carcinoma treated with neoadjuvant chemoradiotherapy and surgery
Source: PLoS One. 2024 Mar 28;19(3):e0300173. doi: 10.1371/journal.pone.0300173 (PMC10977715; doi:10.1371/journal.pone.0300173)
Supplement: Attachment — Submitted filename: Rebuttal letter.doc [file pone.0300173.s003.doc]

**Point-to-point Response to Editor and Reviewers**

We have made revisions in this new version according to your and the reviewers’ comments as follows:

**Response to Editor (Journal requirements):**

We appreciate the valuable comments and suggestions.

**1.** Please ensure that your manuscript meets PLOS ONE's style requirements, including those for file naming.

**Response:** We ensure that our manuscript meets PLOS ONE's style requirements.

**2.** In your Data Availability statement, you have not specified where the minimal data set underlying the results described in your manuscript can be found. PLOS defines a study's minimal data set as the underlying data used to reach the conclusions drawn in the manuscript and any additional data required to replicate the reported study findings in their entirety. All PLOS journals require that the minimal data set be made fully available.

Upon re-submitting your revised manuscript, please upload your study’s minimal underlying data set as either Supporting Information files or to a stable, public repository and include the relevant URLs, DOIs, or accession numbers within your revised cover letter. Any potentially identifying patient information must be fully anonymized.

Important: If there are ethical or legal restrictions to sharing your data publicly, please explain these restrictions in detail. Please see our guidelines for more information on what we consider unacceptable restrictions to publicly sharing. Note that it is not acceptable for the authors to be the sole named individuals responsible for ensuring data access.

We will update your Data Availability statement to reflect the information you provide in your cover letter.

**Response:** We have uploaded the minimal data set underlying the results in a supporting information file, S1 Dataset.

**3.** Thank you for submitting your work to PLOS ONE. Before we can proceed, please report in the Methods section the dates when data were accessed for research purposes. Please note that if this information is not included when your manuscript is resubmitted, it may be rejected.

**Response:** We have reported when data were accessed for research purposes in page 5 line 79-81 of our revised manuscript.

**4.** Please review your reference list to ensure that it is complete and correct. If you have cited papers that have been retracted, please include the rationale for doing so in the manuscript text, or remove these references and replace them with relevant current references. Any changes to the reference list should be mentioned in the rebuttal letter that accompanies your revised manuscript. If you need to cite a retracted article, indicate the article’s retracted status in the References list and also include a citation and full reference for the retraction notice.

**Response:** We ensure that our reference list is complete and correct. There was no change to the reference list in this revised manuscript.

**5.** Additional Editor Comments: The article is clearly written and provides an interesting analysis of the correlation of nodal volume and survival in SCC esophageal cancer. This should represent the basis of a prospective project.

**Response:** We appreciate the valuable comments.

**Response to Reviewers:**

**Reviewer 1:** The manuscript is presented in an intelligible fashion and written in standard English. The data support the conclusions. It is an interesting work. It deserves publication. No changes needed to the article.

**Response:** We appreciate the valuable comments.

**Reviewer 2:** The work seems interesting to me and has a potential impact on clinical practice. Obviously, I would advise the authors to continue with the study perhaps considering a prospective study in order to eventually confirm the reported data.

**Response:** The valuable comments and suggestions are highly appreciated. We will consider prospective studies to validate our findings.

**Reviewer 3:** Interesting work, please review this comment in order to publish.
"PET-CT was performed in cases with indeterminate results on CT or bone scan": risk of target missing should be underlined, it could be interesting to know the rate of locoregional failure among patients undergoing PET for stage assessment.

**Response:** We appreciate the valuable comments. PET-CT was carried out in 45 (42.1%) patients before neoadjuvant CCRT. It was not associated with locoregional failure (p = 0.808), DFS (p = 0.742), and OS (p = 0.804). The paragraphs describing these results have been integrated into page 10 line 181-183, and the relevant data have also been added in Table 1 and Table 3 of our revised manuscript.
